# Pricing combination products: not how but who?

**DOI:** 10.1007/s10198-025-01773-8

**Published:** 2025-04-17

**Authors:** Adrian Towse, Andrew Briggs, Lotte Steuten

**Affiliations:** 1 Office of Health Economics, 2nd Floor Goldings House, Hay’s Galleria, 2 Hay’s Lane, London, SE1 2HB UK; 2 London School of Hygiene & Tropical Medicine, Keppel Street, London, WC1E 7HT UK

**Keywords:** L51, I18

## Abstract

In the last decade progress has been made in identifying solutions to the “technical problem” of attributing the value of combinations between component parts, but not in adapting mechanisms to implement solutions. We propose a way forward to address the “mechanism problem”, arguing that it is essential HTA bodies and/or pricing and reimbursement authorities get actively involved in setting out attribution rules or methods. HTA and pricing/reimbursement authorities have, in essence, adopted one of three strategies: (i) “Do nothing”; (ii) Take a simplistic and arbitrary approach, such as the German law imposing a “haircut” of 20% on the prices of products used in combination or (iii) “Passing the parcel” to the companies and to competition authorities, hoping they will solve the problem for them. Even if a competition law compatible solution is possible, three challenges remain. First, the cost and effort of using it may be too high in relation to any likely gains. Second, the bargaining power of the backbone owner under current HTA / pricing rules is so high that, likely, no solutions that incentivise add-on therapy development will emerge from a process from which HTA bodies absent themselves. Third, most solutions emerging from such a process which give any returns to the add-on likely need the backbone to have a different price (i.e. lower) in combination use as compared to monotherapy use, requiring payer approval for multi-indication pricing. Resolution of the combination challenge thus requires HTA and reimbursement bodies involvement in value attribution.

Payers have found it difficult to reimburse treatment combinations that use more than one on-patent medicine against a background in which combining or sequencing on-patent medicines is becoming increasingly common, notably in oncology [1]. Pipeline analysis indicates that there are many more combination therapies in development [2].

The combination treatment issue is not new. A 2013 report for the UK’s NICE highlighted that add-on products to existing treatments that improved health outcomes could be “not cost-effective at zero price” [3, 4], i.e., even if products were given away by the innovator. A number of papers have articulated the problems and looked at possible ways forward in different jurisdictions [5–12].

There are two elements to the problem: (i) the technical problem: how do we technically divide the value of a combination or sequence of treatments, given the health gain is a joint product of the components; (ii) the mechanism problem: what are the mechanisms needed to implement the technical solution?

In this editorial, we argue that sufficient progress has been made in identifying solutions to the “technical problem” but little in adapting mechanisms to implement solutions. We propose a way forward to address the “mechanism problem”. In particular, we argue that it is essential that payers (via their agents, be they HTA bodies and/or pricing and reimbursement authorities) get actively involved in setting out attribution rules or methods.

In tackling the technical problem, we have, separately, written papers [13–18] which propose solutions to the attribution of value between products, with many similarities. Key challenges include:

* The need to address information challenges about effectiveness if one or more of the on-patent products in the combination does not already have evidence from a monotherapy setting.

* The need to understand whether the combination produces results that are greater or less than the combined separate values of the monotherapies (does “2 + 2 = 5”, or “2 + 2 = 3”?). We can think of this as the degree of additivity. The problem of attribution becomes more acute with sub-additivity, i.e. when “2 + 2 = 3”, and evidence suggests this is likely to be the most common case [19].

Solutions can involve (i) the add-on product receiving the residual value, assuming the backbone product gets at least the same revenues as it currently does; (ii) using a ratio of monotherapy values to share the value of the combined treatments; and (iii) using a weighted ratio for each element of contribution of each product to the combination value, in effect an estimate of “average” marginal value.

Although these solutions have used QALYs as the health numeraire for ease of illustration, there is no reason why these solutions cannot be used in a non-QALY context, such as a Therapeutic Value-Added system of reimbursement. It simply requires using a measure of health, or a composite index, that is relevant to the disease area. In other words, these attribution methods are neutral with respect to the mechanism used to place an overall value or price on the combination. The overall value or price for the combination could be set using, for example, the ASMR framework in France or the AMNOG framework in Germany. Others may come up with new approaches to address attribution issues, but in essence we have technical solutions that work.

The real challenge is the mechanism (or “market design”) challenge [20]. How can a technical solution be implemented in practice? HTA bodies and pricing authorities have, in essence, adopted one of three strategies.

1. “Do nothing”. Evidence from a 2020 OECD survey [21] (p29 Table 2.4.) found that most OECD countries (including most European countries) were not doing anything. They assessed a combination as presented to them. The consequences of doing nothing are, however, two-fold:
  - Where incumbents block use of their products as part of more innovative combinations the returns to add-ons are likely to be low, and so fewer will be developed to be used in combination with existing backbone treatments, reducing health gain for patients.
  - Companies may seek to reduce the bargaining power of the backbone by developing their own backbone products in tandem with their add-ons. According to senior industry leaders, this is already happening. This means that a new combination can be brought to market as the value attribution problem has been internalised within the company. The problem with this solution is that it (1) requires “unnecessary” clinical development including use of limited clinical infrastructure and of a finite pool of patients eligible for inclusion in clinical trial, and (2) involves clinicians having to use an add-on to a new unfamiliar “backbone”. While they are familiar with managing dosing and side-effects of the current monotherapy, the new “backbone” may behave differently.
  2. Take a simplistic and relatively arbitrary approach, such as the German introduction of a “haircut” of 20% on the prices of products used in combination in certain circumstances, approved as part of the 2022 GKV Financial Stabilisation Act. If the add-on has not already been priced as a monotherapy then applying the “haircut” to the price of the backbone in combination use will create headroom for pricing the add-on. However, the arbitrary nature of the intervention is likely to involve “rough justice” and it is very unlikely that the resulting prices will send efficient signals for R&D. What we really want are payer pricing signals to companies that mean add-ons that provide value-for-money in terms of health gain get developed and those that don’t, do not.
2. “Passing the parcel” from the payer to the companies and to the competition authorities. The argument for this approach is that it is up to the (joint) owners of the combination to bring a solution to the payer, and for the competition authorities to find a way (a “safe harbour”) to let them talk to each other (and perhaps share revenues) in a way that does not compromise competition law—given that they are also actual or potential competitors with competing monotherapies or combinations [22]. We can note in this context the 2023 statement from the UK Competition and Markets Authority [23] that it will not prioritise enforcement action against drug firms when they implement a specific ‘negotiation framework’ to make more combination therapies available on the NHS where certain market features are present and particular conditions are met. This is the first official statement from any global competition authority, which provides an important opportunity to explore the feasibility of this route. However, there four potential problems remain with “passing the parcel” to the companies and competition authorities:
  - Designing a solution compatible with competition law may not be possible in many jurisdictions.
  - The resources needed for each company to negotiate solutions for each combination indication in each jurisdiction may far exceed the expected revenues in many situations.
  - The bargaining power of the owner of the backbone under most current HTA / pricing rules is so high that, arguably, no solutions that incentivise add-on therapy development in line with the technical attribution methods we have proposed are likely to emerge from a process from which payers and their agents absent themselves.
  - Even if efficient solutions (or indeed any solutions) were to emerge from such a process (i.e. they give revenues to the add-on that reflect its contribution to the combination) they are likely to involve the backbone having a different price (i.e. lower) in combination use as compared to monotherapy use. This is not, strictly, a problem for the HTA or (perhaps) the pricing and reimbursement body, but it is a challenge for the payer, who has to be able to operationalise indication-based pricing (or permit revenue sharing) to make this happen.

Resolving these problems will require the involvement of the payer. Even if a solution to the competition law problem is found that is not cost/resource prohibitive to companies and rewards add-ons in a way that is dynamically efficient, then implementation in the health care system will still require their intervention.

Many initiatives have been trialled and some are in progress, as countries in Europe and elsewhere have been willing to explore new options. These include [24] the introduction of third parties or trading platforms to facilitate pricing negotiations, and involvement of the HTA body in managing information flows between the negotiating companies [25].

It is our view, however, that payers have to get involved in the technical solutions as well. Seeking to address mechanisms is important, but without payer ownership of the attribution issue, then efficient sustainable mechanism solutions are likely to remain distant. Resolution of the combination challenge therefore requires HTA and reimbursement bodies involvement in value attribution on behalf of their payers.

We now have the technical solutions. Abstinence of involvement on the part of payers becomes ever harder to justify. By all means let’s pursue innovative negotiation mechanisms, but embracing the (technical) attribution issue head on is likely to provide the most efficient solutions, by which we mean the most health gain for patients over time, considering the opportunity cost of scarce health care resources.
